# Loss of phosphoinositide 3-kinase γ decreases migration and activation of phagocytes but not T cell activation in antigen-induced arthritis

**DOI:** 10.1186/1471-2474-11-63

**Published:** 2010-04-07

**Authors:** Michael Gruen, Christina Rose, Christian König, Mieczyslaw Gajda, Reinhard Wetzker, Rolf Bräuer

**Affiliations:** 1 Institute of Molecular Cell Biology, Center for Molecular Biomedicine, Friedrich Schiller University, Jena, Germany; 2 Institute of Pathology, University Hospital, Jena, Germany

## Abstract

**Background:**

Phosphoinositide 3-kinase γ (PI3Kγ) has been depicted as a major regulator of inflammatory processes, including leukocyte activation and migration towards several chemokines. This study aims to explore the role of PI3Kγ in the murine model of antigen-induced arthritis (AIA).

**Methods:**

Development of AIA was investigated in wildtype and PI3Kγ-deficient mice as well as in mice treated with a specific inhibitor of PI3Kγ (AS-605240) in comparison to untreated animals. Inflammatory reactions of leukocytes, including macrophage and T cell activation, and macrophage migration, were studied *in vivo *and *in vitro*.

**Results:**

Genetic deletion or pharmacological inhibition of PI3Kγ induced a marked decrease of clinical symptoms in early AIA, together with a considerably diminished macrophage migration and activation (lower production of NO, IL-1β, IL-6). Also, macrophage and neutrophil infiltration into the knee joint were impaired *in vivo*. However, T cell functions, measured by cytokine production (TNFα, IFNγ, IL-2, IL-4, IL-5, IL-17) *in vitro *and DTH reaction *in vivo *were not altered, and accordingly, disease developed normally at later timepoints

**Conclusion:**

PI3Kγ specifically affects phagocyte function in the AIA model but has no impact on T cell activation.

## Background

Rheumatoid arthritis (RA) is a painful and disabling autoimmune disorder, affecting about one percent of the population in Western countries [1]. As a main indication the disease comprises chronic inflammation of pheripheral joints, resulting in progressive destruction of articular cartilage and bone [2]. Inflamed tissue is characterized by infiltration of leukocytes, pannus formation and occurrence of aggressive synovial fibroblasts [1]. Enhanced expression of several cytokines or matrix metalloproteinases by these cells promotes pathogenicity [reviewed in [3,4]]. Moreover, various chemotactic factors are produced or activated in the joint tissue, recruiting even more leukocytes and exacerbating inflammation [5].

Despite major advantages during the last decade, currently available therapeutic approaches for RA have only partial clinical benefit and are associated with considerable side effects. Treatment strategies include anti-inflammatory or immunosuppressive drugs and biologicals, e.g. antibodies against TNF. Recently, prevention of leukocyte infiltration in inflamed tissue by blocking chemokines or chemokine receptors has also been explored but with limited success [6], possibly due to redundancy, enabling efficient leukocyte responses, even when one particular factor is blocked. Therefore current investigations are directed to the suppression of mutual intracellular signaling pathways shared by multiple chemokines. One prominent protein, integrating chemokine signaling in leukocytes, is PI3Kγ, a G-protein-coupled receptor (GPCR) isoform of phosphoinositide 3-kinases [7]. This enzyme was shown to regulate chemotactic responses of neutrophils, macrophages and T cells to several stimuli, including IL-8, C5a and SDF-1α [8]. Furthermore, PI3Kγ is involved in oxidative burst induction in phagocytes [9,10] and activation of T cells [11,12]. Thus, ablation of PI3Kγ could prevent both, leukocyte infiltration into joints and autoimmune activation. Indeed, Camps *et al*. showed a marked suppression of joint inflammation and reduced tissue destruction by inhibition of PI3Kγ activity in the mouse model of collagen-induced arthritis (CIA) [13]. Our data, presented here, prove a role for PI3Kγ in the early phase of murine antigen-induced arthritis (AIA), which is due to decreased phagocyte infiltration into the joint and reduced macrophage activation. However, PI3Kγ-/- mice showed unaltered inflammation at later time points together with normal T cell responses in this model.

## Methods

### Animals

PI3Kγ-deficient (PI3Kγ-/-) mice were described before [8] and backcrossed to the C57BL/6 background for more than 10 generations. Wildtype and PI3Kγ-/- littermates were raised in the Animal Research Facility, Friedrich Schiller University, Jena, Germany. They were kept under standardized conditions with food and water ad libitum in a 12 hour light/dark rhythm. All animal studies were approved by the local commission for animal protection.

### Antigen-induced arthritis and delayed-type hypersensitivity reaction

Wildtype control and PI3Kγ-/- mice, age 7-8 wk, were immunized s.c. at 21 and 14 days before AIA induction with 100 μg of methylated BSA (mBSA; Sigma, Deisenhofen, Germany), dissolved in 50 μl 0.9% NaCl and emulsified with an equal volume of CFA (Sigma), supplemented with 2 mg/ml *Mycobacterium tuberculosis *(Difco). Additionally to immunization with mBSA/CFA, 5 × 10^8 ^heat inactivated *Bordetella pertussis *germs (Chiron-Behring, Liederbach, Germany) were administered i.p. Arthritis was induced by intraarticular inoculation of 100 μg mBSA in 25 μl normal saline solution (0.9% NaCl) in the right knee joint (day 0), leading to development of severe acute synovitis associated with subsequent cartilage and bone erosion in the arthritic joints. At days 2, 7 or 21, animals were sacrified by cervical dislocation. Knees were dissected for histology, spleen and lymph node cells were isolated, and macrophages were prepared by rinsing peritoneal cavitiy with ice-cold PBS (see below). For treatment experiments, PI3Kγ inhibitor AS-605240 (Enzo, Loerrach, Germany) was dissolved in 0.9% NaCl and orally administered every 12 hours for 7 days with 50 mg/kg of body weight in a total volume of 200 μl. Delayed-type hypersensitivity (DTH) reaction in the ear was induced by intradermal injection of 5 μg of mBSA in 10 μl 0.9% NaCl at day 7 of AIA.

### Clinical assessment of AIA and DTH

Knee swelling was measured during the devolution of AIA at definite time points using an Oditest caliper (Kroeplin, Schlüchtern, Germany). DTH reaction was estimated by increase of ear thickness 24 and 48 h after challenge. For histopathological exploration, knee joints were fixed in 4.5% formaldehyde, decalcified with EDTA, embedded in paraffin and cut into 5-μm-sections. Serrations were stained with haematoxylin and eosin and evaluated in a blinded manner according to a histological scoring system ranging from 0 to 3 (0: no, 1: mild, 2: moderate, 3: severe alterations). The amount of fibrin exudation, the relative number and density of granulocytes in synovial membrane and joint space allowed grading of the acute inflammatory reaction, the relative number and density of infiltrating mononuclear leukocytes in the synovial membrane, the degree of synovial hyperplasia, the extent of infiltration and fibrosis in the periarticular structures allowed grading of chronic inflammation. The extent of damage of the cartilage surface and bone structures was also evaluated on a scale of 0-3, where 0 = no damage, 1 = mild destruction, 2 = moderate destruction, and 3 = severe destruction of cartilage and bone (extensive area of chondrocyte death and cartilage destruction, deep invasive bone erosions).

### Immunohistochemical examinations

Cryosections of knee joints were fixed in acetone, blocked with 4% milk/Tris, and stained with 10 μg/ml primary rat mAbs directed against mouse Mac-1 (macrophages and neutrophil granulocytes; Medac, Wedel, Germany) or Gr-1 (neutrophil granulocytes; BD Biosciences, Heidelberg, Germany) for 1 h at 22°C, followed by incubation with biotinylated secondary goat anti-rat IgG Abs (Dako, Glostrup, Denmark). Preparations were re-fixed in 1% formalin, and developed with diaminobenzidine (Sigma) in 0.03% H_2_O_2 _after blocking endogenous peroxidase activity with 0.3% H_2_O_2 _in 0.1 M NaN_3_. Sections were counterstained with Mayer's hematoxylin.

### Peritoneal macrophage activation

Macrophages were isolated at different time points after arthritis induction by peritoneal lavage of three mice with 7 ml ice-cold phosphate buffered saline containing 5 IU/ml heparin (Liquemin N 2000, Roche, Grenzach-Whylen, Germany) [14]. Cells were cultured for 24 hours *ex vivo *in RPMI1640 medium supplemented with 10% FCS without further restimulation; supernatants were collected and cytokines analyzed by ELISA (see T cell activation). Primary antibodies for IL-1β and IL-6 and biotin-labeled secondary antibodies were purchased from BD Biosciences. Nitric oxide production was measured by Griess reagent as described previously [15].

### Migration assay

For *in vitro *chemotaxis, 10^6 ^macrophages/ml were applied to the upper compartment of transwell chambers (Corning, Wiesbaden, Germany; 5 μm pore size) and 600 μl of medium with or without C5a were added to the lower compartment. After 20 h incubation, membranes were excised, upper sides were wiped clean and cells on the lower side stained with cristal violet and counted microscopically. PI3Kγ inhibitor AS-605240 was applied at a concentration of 20 μM.

### Akt phosphorylation

One million macrophages were starved for 4 hours, restimulated with C5a for 5 min, lysed in Laemmli buffer and subjected to SDS-PAGE and immunoblotting. Antibodies were purchased from Cell Signalling (Frankfurt, Germany; Akt, phospho-Akt) and Sigma (β-actin). Secondary HRP-coupled anti-mouse and anti-rabbit antibodies were from KPL (Gaithersburg, USA).

### T cell activation

Single cell suspensions were prepared from draining lymph nodes and spleen and cultured at 10^6^/ml in RPMI 1640 medium containing 10% FCS, 2 mM sodium pyruvate, 10 mM HEPES, 15 μg/ml L-glutamine, 5 μg/ml streptomycin, 5 U/ml penicillin (Invitrogen, San Diego, USA), 5 × 10^-5 ^M β-mercaptoethanol (Sigma) in a humidified atmosphere at 37°C, 5% CO_2 _in the presence of 25 μg/well mBSA or 2 μg/well of plate-bound anti-CD3 antibodies from 1452C11 hybridoma cell supernatant. Supernatants were harvested after 42 h and analyzed for levels of secreted cytokines using standard sandwich ELISA procedures as previously described [16]. Primary and biotin-labeled secondary Abs for IL-2, IL-4, IL-5, IL-17, IFNγ, and TNFα were purchased from BD Biosciences. Detection limit was 10 pg/ml for all ELISAs used.

### Statistical analysis

Differences between the groups were evaluated using nonparametric Mann-Whitney *U*-test and considered statistically significant with p < 0.05. All calculations were performed by means of the SPSS software package (v.10.0.5). Outliers were identified using Grubbs-test. Data are shown as arithmetic mean and SEM.

## Results

### Clinical symptoms of AIA are decreased in PI3Kγ-deficient mice

**Figure 1:**
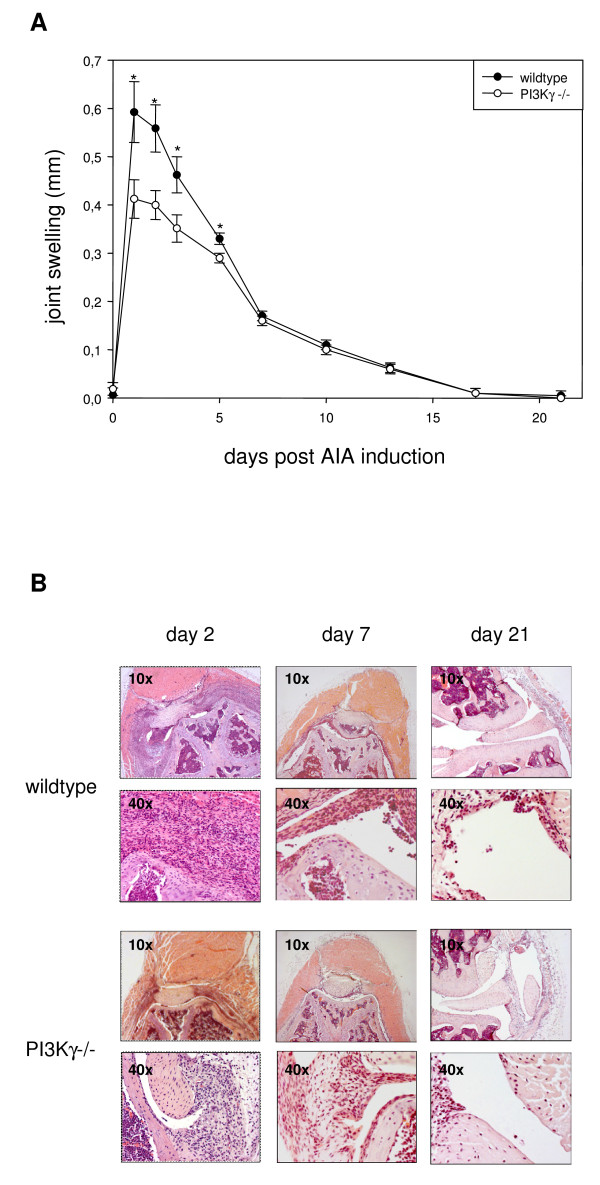
**Clinical manifestation of early AIA is decreased in PI3Kγ-/- mice**. AIA was induced in C57BL/6 wildtype or PI3Kγ-/- littermates (n = 8) at day 0. (A) Joint swelling was measured during three weeks. Mean and SEM are shown, *: statistically significant difference, p < 0,05. (B) Mice were sacrificed at the indicated time points, histological sections were taken and evaluated microscopically (see table 1).

After arthritis induction, wildtype and knockout mice developed rapid inflammation, indicated by acute joint swelling. Knee diameter reached its maximum in both strains at day 1 and declined afterwards. In PI3Kγ-/- mice the swelling response at day 1 was markedly alleviated compared to wildtype controls (figure 1A). This difference was evident for the next days and disappeared at approximately day 7. Afterwards, joint diameter decreased further, declining to baseline values at day 17 for both strains.

At days 2, 7, and 21, mice were sacrificed, histological sections were taken and evaluated for arthritis score (figure 1B and table 1). Paralleling the joint swelling, significant differences between wildtype and PI3Kγ-/- mice were found at day 2, while disease scoring at later timepoints was essentially identical.

**Table 1:** Histological disease scoring of wildtype and PI3Kγ-/- mice during AIA (n = 8, mean ± SEM)

	day 2		day 7		day 21	
	wt	**PI3Kγ**-/-	wt	**PI3Kγ**-/-	wt	**PI3Kγ**-/-
acute inflammation	**1,6 ± 0,5**	**0,7 ± 0,5**	1,0 ± 0,4	0,8 ± 0,5	0,2 ± 0,2	0,2 ± 0,2
chronic inflammation	**0,7 ± 0,3**	**0,1 ± 0,1**	1,6 ± 0,5	1,4 ± 0,5	1,0 ± 0,4	0,8 ± 0,3
destruction	0,5 ± 0,2	0,3 ± 0,3	0,8 ± 0,1	0,7 ± 0,2	0,5 ± 0,2	0,4 ± 0,2

total score	**2,8 ± 1,0**	**1,1 ± 0,9**	3,4 ± 1,0	2,9 ± 1,2	1,7 ± 0,8	1,4 ± 0,7

bold: significant differences between groups (p < 0.05)

### Reduced activation and migration of phagocytes

**Figure 2:**
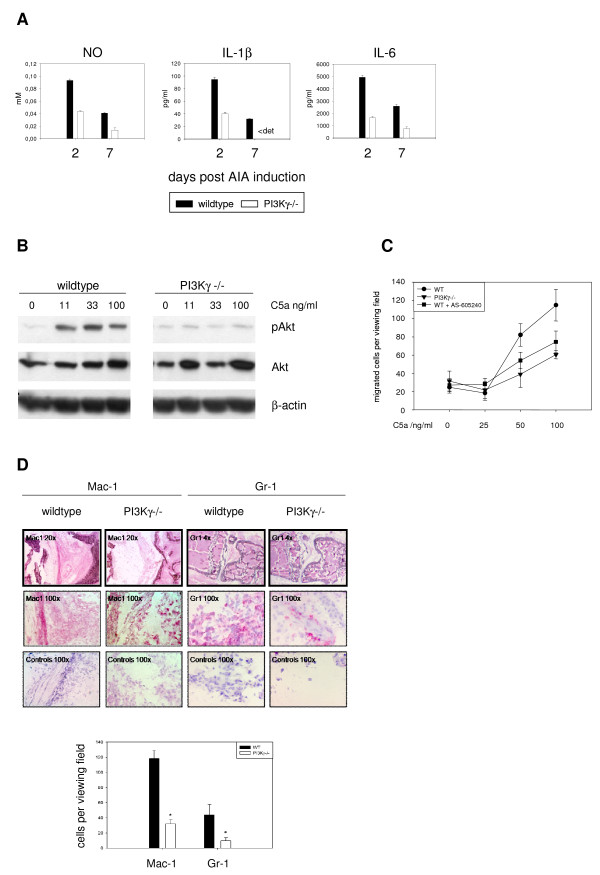
**Phagocyte functions are impaired in PI3Kγ-/- mice**. Peritoneal macrophages of three wildtype and three knockout littermates were isolated at different time points, cells were pooled and used for *in vitro *experiments. (A) Production of NO, IL-1β, and IL-6 was measured after 24 hours culture without restimulation. Mean and SEM of triplicates are shown for one of two experiments. < det: below detection limit (10 pg/ml) (B) Macrophages isolated at day 2 of AIA were starved for 4 hours, restimulated for 5 min with C5a, as indicated, lysed and subjected to immunoblotting for Akt, phospho-Akt (pAkt), and β-actin. (C) Macrophages isolated at day 2 of AIA were seeded into transwell plates and migrated towards different concentrations of C5a for 20 hours. Afterwards, membranes were washed and cells at the lower side were stained and counted microscopically. One of two experiments is shown (mean and SEM). (D) Joint sections at day 2 of AIA were prepared and stained with Mac-1 (macrophages, neutrophils) or Gr-1 (neutrophils) specific antibodies (upper panel) and positive cells were counted (lower panel, mean and SEM of 6 animals). *: statistically significant difference, p < 0.05.

Maximal macrophage activation has been reported to occur during the first days of AIA [17]. We speculated, that amelioration of clinical symptoms during these days might reflect impaired macrophage activity in the PI3Kγ-/- mice. Peritoneal macrophages were isolated from animals at days 2 or 7 of AIA and tested *ex vivo *for production of NO, IL-1β, and IL-6 without further restimulation. None of these molecules could be detected in macrophages of naïve mice (data not shown), while cells of arthritic mice produced all three mediators. At all timepoints investigated, PI3Kγ-/- macrophages synthesized significantly lower levels of NO, IL-1β, and IL-6 (figure 2A).

PI3Kγ is also known to control migration of phagocytes. Thus, we investigated peritoneal macrophages at day 2 for their *in vitro *reaction to complement factor C5a, a known chemotactic agent in AIA which signals via PI3Kγ [8]. After starvation and restimulation with different concentrations of C5a, phosphorylation of Akt (pAkt), a major downstream target of PI3K, was investigated. Wildtype macrophages showed pronounced phosphorylation in this experiment. In contrast, C5a was not able to induce pAkt in PI3Kγ-deficient cells (figure 2B). Moreover, in a transwell chemotaxis assay, wildtype cells were much more responsive to C5a stimulation than PI3Kγ-deficient macrophages. By adding the selective PI3Kγ-inhibitor AS-605240 [13] to the assay, migration of wildtype cells could be decreased nearly to the level, observed with PI3Kγ-/- macrophages (figure 2C).

Finally, immunohistochemical analysis of inflamed joints revealed a markedly reduced infiltration of PI3Kγ-deficient macrophages and neutrophils to the inflamed tissue *in vivo *(figure 2D). We conclude that loss of PI3Kγ reduces activity and migration of innate immune cells at early phases of AIA.

### T cell reactions are largely unaltered in PI3Kγ-/- mice

**Figure 3:**
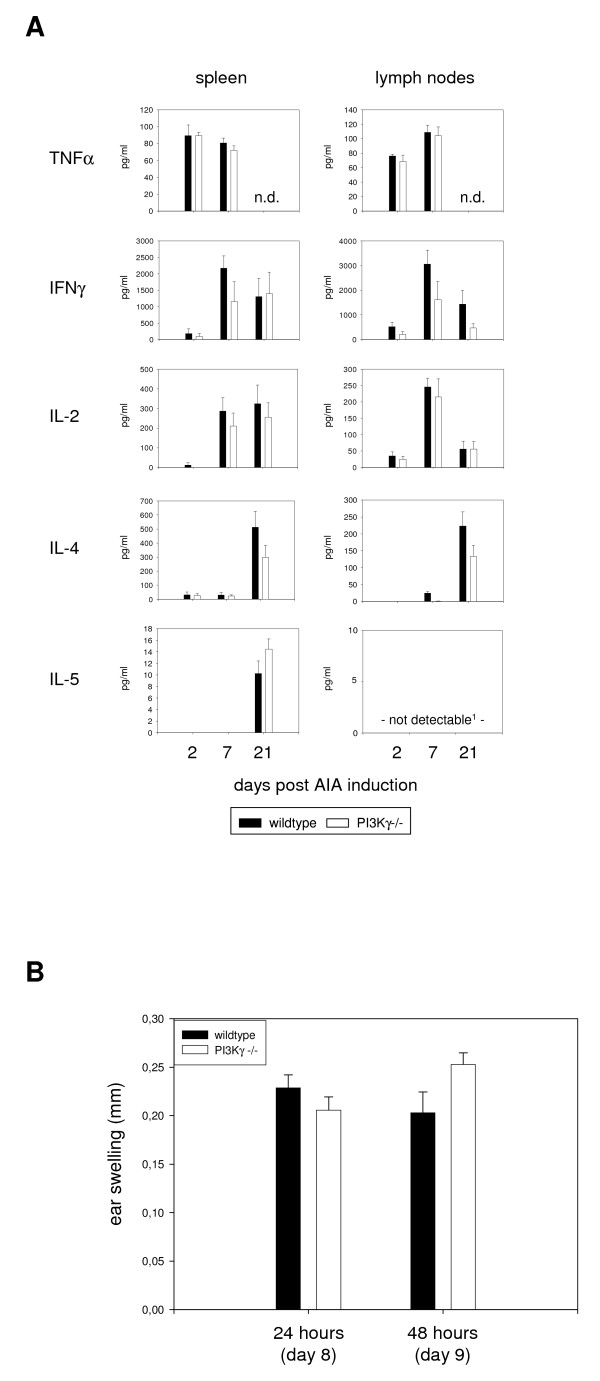
**T cell responses are normal in PI3Kγ-/- mice**. (A) Splenocytes or cells of draining lymph nodes were isolated and restimulated in vitro with mBSA for 24 hours. Supernatants were collected and cytokine production was measured by ELISA (n = 6, one of two experiments shown). ^1^Two outliers were eliminated for day 7 (Grubbs test). (B) DTH reaction was induced at day 7 and ear swelling was determined 24 and 48 hours later.

Clinical markers of acute inflammation and markers of macrophage activity passed through a maximum at day 2 (table 1 and figures 1 and 2). In contrast, chronic inflammatory reactions further increased later on. Moreover, knee swelling and histological scoring were comparable between wildtype and knockout animals at days 7 and 21 (figure 1 and table 1). This implicated pathogenic processes independent of PI3Kγ. Since AIA depends on T helper cells to a large extend [18], we tested the T cell activation in the knockout mice. Splenocytes or cells from draining lymph nodes were isolated at days 2, 7, and 21 and restimulated *in vitro *either polyclonally with anti-CD3-antibodies or specifically with mBSA. Culture supernatants were collected and TNFα, IFNγ, IL-2, IL-4, and IL-5 were measured. There were no significant differences observed between either anti-CD3-stimulated (data not shown) or mBSA-stimulated wildtype and PI3Kγ-deficient cells (figure 3A). At day 2, IL-17 was quantified additionally in the supernatants. Cells stimulated with mBSA did not secrete detectable quantities of this cytokine (data not shown), while after activation with anti-CD3-antibodies comparable amounts of IL-17 were produced by wildtype and PI3Kγ-deficient cells (table 2). Cells of naïve mice did not secrete measurable amounts of cytokines (data not shown).

**Table 2:** IL-17 content in supernatants of anti-CD3-stimulated cell cultures (n = 5, mean ± SEM)

	IL-17 (pg/ml)	
	wt	**PI3Kγ**-/-
spleen	68 ± 54	67 ± 49
lymph nodes	443 ± 61	318 ± 69

To further investigate T cell immune reactions *in vivo*, a delayed-type hypersensitivity test (DTH) was performed at day 7 and ear swelling was measured 24 and 48 hours later (figure 3B). No differences between the strains could be detected, again pointing to normal T cell responses in the PI3Kγ-/- mice.

### Pharmacologic inhibition of PI3Kγ reduces joint swelling and macrophage activity in early AIA

**Figure 4:**
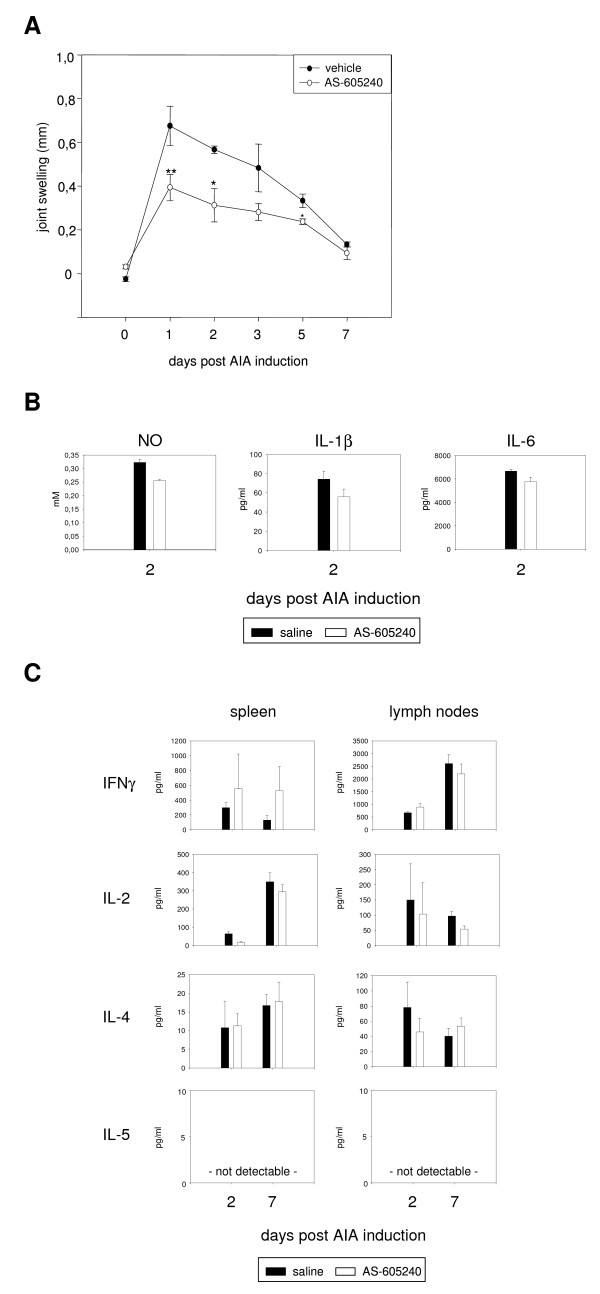
**Pharmacologic inhibition of PI3Kγ alleviates joint swelling and macrophage activity**. Wildtype C57BL/6 mice were immunized with mBSA, AIA was induced and animals randomly assinged to two groups, receiving either AS-605240 or vehicle (saline) for 7 consecutive days. (A) Knee swelling was measured as a clinical marker of AIA. n = 5, *: p < 0.05, ** p < 0.01 (B) Peritoneal macrophages were isolated at day 2 of AIA, cultured for 24 hours without restimulation and production of effector molecules was measured in the culture supernatant (mean and SEM of triplicates). (C) Splenocytes and lymph node cells were isolated at day 2 and 7 of AIA, cultured for 24 hours in the presence of mBSA and cytokines were measured in the supernatants.

Our data implicate specific functions of PI3Kγ in phagocytes during acute inflammatory responses. Nevertheless, the observed alleviation of clinical effects could also be caused by a priming defect during the immunization phase (days -21 to 0). To clarify this and mimick a therapeutic intervention, we again used the inhibitor AS-605240. Wildtype C57BL/6 mice were immunized with mBSA and randomly assigned to two groups at day 0. One group was orally treated with AS-605240 (50 mg/kg in PBS) twice a day for 7 days and the other group received an equal volume of 0.9% saline. Joint swelling and arthritis scores are shown in figure 4A and table 3. The results reflected a similar situation as found with PI3Kγ-/- animals. Joint swelling was markedly reduced in treated mice during days 1 to 5 and was comparable to controls afterwards. Arthritis scores were decreased at day 2 in the group receiving AS-605240, although this effect was not statistically significant. However, at day 7, joint swelling and histological scoring were essentially identical between both groups. In line with these clinical parameters, peritoneal macrophage activity was decreased by AS-605240 treatment at day 2 (figure 4B). With an average reduction of about 20%, production of NO, IL-1β, and IL-6, was not as strongly inhibited in this experiment, as was observed with PI3Kγ-/- mice (compare to figure 2A). Migration of wildtype macrophages towards C5a could be inhibited by AS-605240 (see figure 2C). Cytokine production by T cells was not influenced by the inhibitor at all (figure 4C). These results demonstrate the potential of AS-605240, especially to alleviate clinical signs of acute AIA (joint swelling) and, once again, prove that chronic inflammatory processes in the AIA model are independent of PI3Kγ.

**Table 3:** Histological disease scoring of wildtype mice treated with the specific PI3Kγ inhibitor AS-605240 during AIA (n = 5, mean ± SEM)

	day 2		day 7	
	control	AS-605240	control	AS-605240
acute inflammation	3,0 ± 1,0	1,9 ± 0,8	2,5 ± 1,0	2,5 ± 0,8
chronic inflammation	1,9 ± 0,6	1,4 ± 0,6	2,2 ± 0,7	2,2 ± 0,4
Destruction	1,1 ± 0,1	0,8 ± 0,2	1,3 ± 0,4	1,0 ± 0,3

total score	6,0 ± 1,1	4,1 ± 0,6	6,0 ± 0,6	5,7 ± 0,8

## Discussion

Our study aimed at elucidating the role of PI3Kγ in the mouse model of AIA, which resembles many aspects of RA in humans. PI3Kγ is known to control various effector functions of leukocytes, including migration (e.g. extravasation from blood to tissue), oxidative burst, cytokine production and phagocytosis [8-10,19,20]. This suggests the kinase as a possbile exacerbating factor in autoimmune disorders, including RA. Accordingly, in the AIA model genetic or pharmacologic inactivation of PI3Kγ results in considerably diminished manifestation of early disease. Joint swelling two days after arthritis induction was only about 60% of the controls and histological scoring in the knockouts revealed only about 40% disease intensitiy compared to wildtype animals. These findings are in line with studies on PI3Kγ in mouse models of CIA or serum-induced arthritis [13,21] and underline the importance of this molecule in acute inflammatory processes. Quite surprisingly, in the AIA model at later time points, PI3Kγ-/- mice revealed a fully developed disease with knee swelling and histological scoring being comparable between controls and knockouts. These observations were in contrast to both other studies on PI3Kγ in arthritis [13,21] but may be explained by different requirements for T cells among the three models. Randis *et al*. [21] induced arthritis by serum transfer from K/BxN mice to PI3Kγ-/- on a C57BL/6 background, a model that was reported to be independent of lymphocytes [22]. Likewise, T cells are dispensable for the development of anti-CII antibody-induced arthritis [23], one of the models investigated by Camps *et al*. [13]. However, the same group also studied CIA, which requires T cell activation, and found considerably decreased disease symptoms upon treatment of mice with AS-605240. The reason for this discrepancy is not completely clear, but most probably, the relative contribution of innate immunity to CIA is much stronger than in the later (chronic) phases of the AIA model. This might be reflected by the different pattern of paw and knee swelling: while paw thickness in the CIA model steadily increases during the whole observation period, the knee swelling in AIA has a maximum at day 1 and decreases thereafter.

Macrophage activation peaked at day 2 in our experiments, while main T cell responses were observed at days 7 and 21. From this, we hypothesized, that loss of PI3Kγ mostly inhibits phagocyte activation and function, whereas T cells are less or even not affected. Our data *in vivo *and *in vitro *support this hypothesis: Effector production by peritoneal macrophages (NO, IL-1β, IL-6) *ex vivo *was clearly decreased in PI3Kγ-/- mice during the course of AIA. It is important to note, that cytokine production in this experiment was not induced by external stimuli, specifically targeting GPCR receptors and PI3Kγ, but rather reflected the state of *in vivo *macrophage priming. Thus, our results extend data of several groups, investigating PI3Kγ-dependent activation of macrophages *in vitro *[9,10,20]. In our experiments, C5a induced phosphorylation of Akt in wildtype macrophages *in vitro*, while no phosphorylation could be observed in PI3Kγ-/- cells. This is in line with previous reports [8,13] and contradicts to some extend a recent study, showing concerted and partially redundant action of PI3Kγ and PI3Kβ isoforms downstream of GPCR [24]. These issues require further investigation and clarification. Additionally, macrophage migration towards C5a *in vitro *and infiltration of both, neutrophils and macrophages to the inflamed joint *in vivo *were diminished in the PI3Kγ-/- mice and migration of wildtype macrophages could be blocked *in vitro *by using AS-605240. Taken together, innate immunity seems to be largely impaired by loss of PI3Kγ, causing the marked alleviation of clinical symptoms at early days of AIA.

On the other hand, our experiments revealed no changes in T cell activation of PI3Kγ-/- mice. Neither cytokine production *in vitro *(TNFα, IFNγ, IL-2, IL-4, IL-5, IL-17) was significantly altered, nor was the DTH reaction *in vivo*. Those results were somewhat surprising, considering several reports on involvement of PI3Kγ in T cell maturation, migration and activation [25-28]. However, most of those results were obtained *in vitro*, while *in vivo *data are less clear. Most groups investigated an acute activation of T cells by anti-CD3-antibodies *in vitro *or migration of naïve T cells towards SDF-1α in a time frame of several hours. Contrary, the immunization period of the AIA model (3 weeks) might provide sufficient time for efficient T cell priming and mounting of effector function. Moreover, in two models of systemic lupus erythematosus, PI3Kγ was shown to control the CD4+ memory T cell reservoir mostly in the context of a second genetic alteration (MRL-*lpr *or p65^PI3K^Tg), while single-targeted PI3Kγ-inhibition revealed only modest phenotypes [27,29]. Additionally, although defective T cell differentiation can be detected in newborn PI3Kγ-deficient mice, differences decrease in mice of about one month of age [12]. Those data suggest an at least partial compensation of PI3Kγ *in vivo*, dependend on the microenvironment and activation status of the T cell. This premise is supported by recent studies of Ward and colleagues on human T lymphocytes. The activation status or even culture conditions strongly influenced requirement of PI3Kγ for migration in these experiments [30,31].

The DTH reaction at 24 and 48 hours post challenge was unchanged in PI3Kγ-/- mice (days 8 and 9 of AIA). This again demonstrates a largely unaltered T cell response and suggests a compensation of deficient signaling reactions in PI3Kγ-/- T cells by other proteins. Although defective development of thymocytes or CD4 memory T cells as described by Rodriguez-Borlado *et al*. [12] and Barber *et al*. [27] was not addressed by our experiments, this does not seem to primarily influence disease development.

The effects of genetic ablation of PI3Kγ could be mimicked largely by using the inhibitor AS-605240, specific for PI3Kγ. This compound was able to markedly decrease macrophage activation and joint swelling during the first days (acute phase) of AIA, in accordance to Camps *et al*. [13]. Efficacy of AS-605240 in both models points to a possible impact in human rheumatoid arthritis too and asks for further development of this drug candidate towards clinical use. Although T cell activation was not influenced by the inhibitor, its ability to alleviate clinical parameters specifically during early AIA suggests a potential of AS-605240 or other PI3Kγ inhibitors preferentially in therapy of acute exacerbations of the disease, a hallmark of chronic RA in humans.

## Conclusions

In summary, PI3Kγ has its greatest impact on AIA during the first days of the inflammation, when innate immune cells dominate pathogenesis. The enzyme controls production of NO and cytokines by macrophages and migration of macrophages and neutrophils. Deletion or inhibition of PI3Kγ does not influence T cell responses in the AIA model. Accordingly, disease becomes fully developed at later stages, as judged from T cell cytokine production and histological sections of inflamed joints. Thus, influence of PI3Kγ on adaptive immune reactions is rather limited in this model.

## List of abbreviations

AIA: antigen-induced arthritis; CIA: collagen-induced arthritis; mBSA: methylated bovine serum albumin; PI3K: phosphoinositide 3-kinase; RA: Rheumatoid arthritis

## Competing interests

The authors declare that they have no competing interests.

## Authors' contributions

MGr, CR, RW and RB designed research, MGr, CR, CK and MGa conducted experiments, MGr, CR, RW and RB interpreted data, MGr, CR and RB drafted the manuscript, RW and RB raised funding. All authors read and approved the final manuscript.

## Pre-publication history

The pre-publication history for this paper can be accessed here:

http://www.biomedcentral.com/1471-2474/11/63/prepub
